# Anatomical Vascular Differences and *Leishmania*-Induced Vascular Morphological Changes Are Associated with a High Parasite Load in the Skin of Dogs Infected with *Leishmania infantum*

**DOI:** 10.3390/pathogens13050371

**Published:** 2024-04-30

**Authors:** Francini N. Ribeiro, Tainã L. de Souza, Rodrigo C. Menezes, Lucas Keidel, João Paulo R. dos Santos, Igor J. da Silva, Marcelo Pelajo-Machado, Fernanda N. Morgado, Renato Porrozzi

**Affiliations:** 1 Laboratório de Protozoologia, Instituto Oswaldo Cruz—FIOCRUZ, Rio de Janeiro 21040-360, Brazil; francini.nribeiro@outlook.com (F.N.R.); tls.desouza@gmail.com (T.L.d.S.); 2 Laboratório de Imunoparasitologia, Instituto Oswaldo Cruz—FIOCRUZ, Rio de Janeiro 21040-360, Brazil; 3 Laboratório de Pesquisa Clínica em Dermatozoonoses em Animais Domésticos, Instituto Nacional de Infectologia Evandro Chagas—FIOCRUZ, Rio de Janeiro 21040-360, Brazil; rodrigo.menezes@ini.fiocruz.br (R.C.M.); lucas.keidel@ini.fiocruz.br (L.K.); 4 Laboratorio de Medicina Experimental e Saúde, Instituto Oswaldo Cruz—FIOCRUZ, Rio de Janeiro 21040-360, Brazil; joaopaulorsantos@gmail.com (J.P.R.d.S.); silva.igorj@gmail.com (I.J.d.S.); marcelo.pelajo@fiocruz.br (M.P.-M.); 5 Brazilian National Institute of Science and Technology on Neuroimmunomodulation, Instituto Oswaldo Cruz—FIOCRUZ, Rio de Janeiro 21040-360, Brazil

**Keywords:** visceral leishmaniasis, vascularization of skin, dogs, *Leishmania infantum*, inflammatory reaction

## Abstract

Canine visceral leishmaniasis (CVL), caused by the protozoan *Leishmania infantum*, affects several organs, including the skin. Dogs are considered the major domestic reservoir animals for leishmaniasis, and through their highly parasitized skin, they can serve as a source of infection for sandfly vectors. Therefore, studies of the skin parasite–host relationship can contribute to the understanding of the infectious dissemination processes of parasites in the dermis and help to identify targets for diagnosis and treatment. Thus, the aim of this study was to evaluate the association of anatomical vascular differences and *Leishmania*-induced vascular morphological changes with clinical signs and parasite load by analyzing the ear and abdominal skin from dogs naturally infected with *L. infantum*. Paired samples of ear and abdominal skin from *L. infantum*-positive dogs (*n* = 26) were submitted for histological and immunohistochemistry analyses. The ear skin samples showed a more intense and more diffusely distributed granulomatous inflammatory reaction, a higher number and larger diameter of blood vessels, increased parasite load, higher expression of VEGF+ (vascular endothelial growth factor) and MAC 387+ (calprotectin) recently infiltrating cells, and more intense collagen disruption compared to the abdominal skin samples. Intracellular amastigotes were observed in blood vessels and inside endothelial cells and were diffusely distributed throughout the dermis in the ear skin samples. The NOS2/MAC387+ cell ratio was lower in the ear skin samples than in those of the abdomen, suggesting that in the ear dermis, the inflammatory infiltrate was less capable of producing NO and thereby control the parasite load. Together, these findings indicate how parasites and immune cells are distributed in the skin and suggest an important role for dermal vascularization in cellular influx and thereby in parasite dissemination through the skin of naturally infected dogs.

## 1. Introduction

Canine visceral leishmaniasis (CVL) is a zoonotic disease for which the major etiologic agent is *Leishmania infantum* [1]. In the urban environment, dogs are considered the main reservoirs of the parasite due to the impact of these animals in maintaining the transmission cycle [2]. Parasitic dermotropism in dogs results in a high parasite load in the skin that is then available to the sandfly vector [3]. During natural leishmaniasis infections, the immune response begins in the skin, where the first wave of cytokines and chemokines appears, attracting several cell types from vessels to the injury site and directing the inflammatory response to various profiles [4,5]. Skin lesions in dogs affected by visceral leishmaniasis have been frequently observed and largely discussed in the literature [6,7,8,9,10]. Nunes et al. (2018) [6] demonstrated a high frequency (66.7%) of detection of parasitic DNA in the skin of infected dogs. The skin parasite load correlated with macrophage infiltration and an increase in TLR-2, NOS2, IL-10, and TNF-α expression, and the xenodiagnosis showed that this increase in parasite load in the skin correlated with an increased risk of transmission to sandflies [11]. The parasite load in the skin, as well as the inflammatory pattern in the dermis, varies according to the anatomical region in which they are observed [7].

During inflammatory processes, the influx of immune cells into the injury site results in reduced tissue oxygen levels due to the high O2 demand of these cells [12]. Low oxygen levels are closely related to the regulation of vascular endothelial growth factor (VEGF) mRNA expression and a reduction in NOS2 expression by myeloid cells [12]. VEGF expression induces angiogenesis of blood and lymphatic vessels by promoting the proliferation of vascular endothelial cells and consequent expansion of the capillary network, and it is considered an important factor involved in the inflammatory response [13]. Studies regarding *Leishmania major* have shown that the presence of the parasite in the skin promotes local vascular remodeling through the high expression of VEGF-A and VEGF receptor-2 (VEGFR-2) and consequent proliferation of blood and lymphatic endothelial cells [14]. Thus, *Leishmania* infections result in changes in the morphology of vascular networks in the skin [14].

The head of dogs presents more blood vessels than the abdomen [15]. The vascularity of the skin is divided into three segments: the superficial plexus, the cutaneous plexus, and the subdermal plexus. In dogs, the subdermal plexus is formed by branches of direct cutaneous vessels parallel to the dermis. This difference distinguishes the vascularization of dog skin from that of human skin, where the subdermal plexus is formed from branches of musculocutaneous vessels in perpendicular orientations [16]. In addition, dogs have intense collateral blood flow to adjacent areas of the skin due to the extensive connections between these vascular plexuses [16]. During *L. infantum* infection, the parasite load has been observed to be associated with the dermal vascular plexuses of dogs, suggesting hematogenous spread of the parasite [17,18]. Therefore, due to the greater collateral vascularity in dog skin and the extensive communication between vascular plexuses, the dissemination of amastigotes and the parasite load could increase. The dissemination of *Leishmania (Viannia)* in immune cells migrating from lymphatic vessels has been demonstrated in a murine model [19]; however, the dissemination of *L. infantum* through migrating cells from blood vessels and vascular alterations in the skin of dogs has not been completely elucidated. Therefore, the aim of this study was to evaluate the association of anatomical vascular differences and *Leishmania*-induced vascular morphological changes with clinical signs and parasite load by analyzing the ear and abdominal skin from dogs naturally infected with *L. infantum*.

## 2. Materials and Methods

### 2.1. Animals and Clinical Assessment

Twenty-six dogs with a confirmed diagnosis of CVL by DPP^®^ (Dual Path Platform rapid test for canine Leishmaniasis) and ELISA-Leishmaniasis (Bio-Manguinhos/Fiocruz, Rio de Janeiro, Brazil) submitted for euthanasia by the zoonosis control service in the period from 2009 to 2015, were included in the study. The animals included in this study are part of the group of animals evaluated in the study by Cavalcanti et al. (2015) [10]. *L. infantum* infection identification was confirmed by multilocus enzyme electrophoresis (MLEE) on all strains isolated by the National Reference Laboratory for *Leishmania* Typing at the Instituto Oswaldo Cruz (LRNTL/IOC). Before euthanasia, the dogs were clinically evaluated by two veterinarians, and six characteristic clinical signs of CVL were considered: dermatitis, onychogryphosis, ophthalmic abnormalities, body condition, alopecia, and lymphadenomegaly. Body condition was graded as 0 (ideal: ribs easily palpable with little fat, waist easily seen from above and abdominal silhouette evident), 1 (thin: ribs easily palpable, can be visible, no palpable fat, top of lower back is visible, pelvic bones prominent, waist silhouette and abdomen apparent), 2 (very thin: ribs, lumbar and pelvic bones are readily visible, absence of palpable fat, evidence of other bony prominences and minimal muscle loss), and 3 (extremely thin: all bony structures are prominent and evident from a distance, no noticeable body fat and severe loss of muscle mass). Each clinical sign was evaluated according to its intensity as 0 (absent), 1 (mild), 2 (moderate), or 3 (severe) points (adapted from Quinnell et al. (2001)) [20]. The sum of the points scored by each animal determined its respective clinical score, and the animal could have a low (0 to 2), medium (3 to 6), or high clinical score (7 to 18). Based on clinical information, the dogs were selected as a convenience sampling representing extremes of the disease. They were divided into two groups for this study: group 1—13 subclinical dogs (clinical score = 0), and group 2—13 clinically affected dogs with high clinical scores (clinical score > 7) that presented dermatological changes.

### 2.2. Histopathologic Analysis

Twenty-six ear skin samples and the same number of paired abdominal skin samples were collected during necropsy. In clinically affected dogs with skin lesions in these sites, samples were collected from areas adjacent to the injured skin. The samples were fixed in 10% buffered formalin, embedded in paraffin, cut into 5 µm thick sections, and mounted on glass slides for subsequent histopathological analysis. The samples were stained with hematoxylin and eosin (HE) and analyzed by light microscopy (Nikon Eclipse E400—Tokyo, Japan). When present, the inflammatory infiltrate was analyzed according to its distribution (perivascular or diffuse) and intensity (absent to mild and moderate to severe) and the presence or absence of granulomatous reactions. Dermal collagen disruption was analyzed in tissues stained with Picrosirius Red. A qualitative analysis was performed regarding the regularity of collagen deposition in the dermis: 1 (Regular) for fragments that presented with absent to mild disruption of collagen fibers, and 2 (Disruption) for fragments that presented moderate or intense disruption in collagen fibers, interpreted as a reduction and disorganization of collagen deposition areas. The number and diameter of dermal blood vessels were also evaluated in these samples. All structures in each tissue section that presented a morphology compatible with blood vascular endothelium were considered for measurement. The vascular diameter and number of vessels were quantified per microscopic field using ImageJ 1.48v software (NIH, Bethesda, MD, USA). The analyses were performed by a single blinded person and confirmed by sampling by a second person.

### 2.3. Immunohistochemistry

Paraffin-embedded skin samples were cut into 5 µm thick sections and mounted on silanized slides (silanized slides, DakoCytomation, Carpinteria, CA, USA). The samples were deparaffinized and subsequently hydrated with distilled water. Antigenic retrieval was performed using Trilogy™, followed by endogenous peroxidase blocking (peroxidase blocking reagent, Dako) and nonspecific blocking (0.4% BSA, Sigma, St. Louis, MO, USA). The samples were incubated with polyclonal rabbit anti-*Leishmania* sp. serum obtained in-house according to the protocol described by Quintella et al. (2009) [21], diluted at 1:200 for subsequent parasite load analysis; Calprotectin Monoclonal Antibody (clone MAC-387, Invitrogen Ref: MA1-81381, Waltham, MA, USA) diluted at 1:100 for subsequent cell influx analysis; anti NOS2 Polyclonal Antibody (bs-2072R) diluted at 1:100; and VEGF Monoclonal Antibody (clone VG1, Invitrogen Ref: MA1-16629) diluted at 1:50. Development was performed using SuperPicture™ HRP Polymer Conjugate Broad Spectrum (Life Technologies, Carlsbad, CA, USA) and AEC Concentrated Kit (20×) (GBI Labs, Decatur, GA, USA), followed by counterstaining with Meyer’s hematoxylin. Amastigote count/mm^2^ was calculated for sections immunolabeled for *L. infantum*. Twenty fields (1000× magnification) per sample were randomly selected for counting. The distribution of amastigotes was classified as follows: 1—perivascular for dogs that presented amastigotes located mainly around blood vessels and 2—diffuse for dogs that presented amastigotes diffusely distributed throughout the tissue. MAC 387+, NOS2+, and VEGF+ cells were quantified as percentages and positive cells per mm/2 in 20 random fields (40× magnification). The analyses were performed by a single blinded person and confirmed by sampling by a second person. The photographs were adjusted for brightness and contrast (spread into the dynamic range) using Photoshop.

### 2.4. Immunofluorescence

The paraffin sections were deparaffinized and hydrated in xylol and alcohol solutions and then subjected to antigen retrieval in a pressure cooker (BioSB Tinto Retriever) using citrate buffer (pH 6.0). After this process, the slides were washed in PBS and incubated in a humid chamber overnight at 4 °C with polyclonal rabbit anti-*Leishmania* sp. serum obtained in-house (Quintella et al., 2009) [21] diluted at 1:200 and VEGF monoclonal antibody (clone VG1, Invitrogen Ref: MA1-16629) diluted at 1:200. After incubation, the slides were developed with secondary antibody (Alexa Fluor 488 goat anti-mouse 1/750 or Alexa Fluor 488 goat anti-rabbit 1/750) in a humid chamber for 1 h at 37 °C, washed again with PBS, stained with Evans Blue (1:10,000), counterstained with DAPI (Thermo Fisher, Waltham, MA, USA., cat. 03571), and mounted with ProlongGold (Thermo Fisher, cat P36934). The slides were analyzed using an LSM 710 confocal microscope (Zeiss, Jena, Germany) and recorded with the support of ZEN software (Zeiss, Germany) on the RPT07A microscopy platform of the Oswaldo Cruz Foundation. The analyses were performed by a single blinded person and confirmed by sampling by a second person. The photographs were adjusted for brightness and contrast using Photoshop.

### 2.5. Statistical Analysis

GraphPad Prism^®^ software (version 6.01) was used to perform the statistical analyses. The Shapiro-Wilk test was used to evaluate the normality of the variables. The data were analyzed by the Mann-Whitney test for independent variables with a nonparametric distribution and by an unpaired *t* test for independent variables with a parametric distribution. Comparisons between ear and abdominal skin fragments were analyzed by the Wilcoxon test for variables with a nonparametric distribution and by a paired *t* test for variables with a parametric distribution. Qualitative variables were analyzed by contingency tables using Fisher’s exact test. Each variable analyzed and the corresponding statistical test are indicated in the figures. Values of *p* < 0.05 were considered significant. Significance level was represented as *p*-value summary: * significant, ** very significant, *** or **** extremely significant.

## 3. Results

### 3.1. Ear Skin Exhibited Severe Histopathological Changes Compared with Abdominal Skin

**Figure 1:**
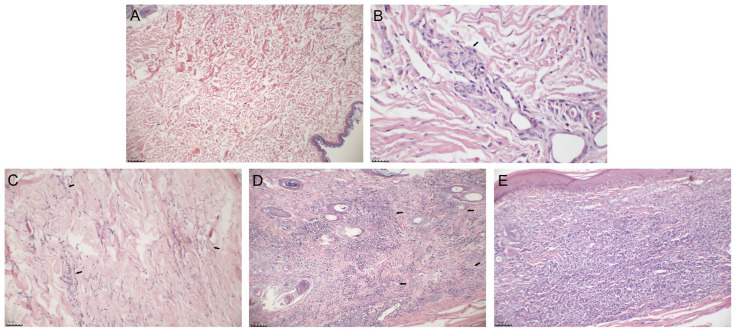
Photomicrographs of the skin of dogs naturally infected with *L. infantum* showing the absence and distribution of inflammatory infiltrate. (**A**) Absent infiltrate in the abdominal skin of a subclinically affected dog. (**B**) Perivascular infiltrate in the ear skin of a subclinically affected dog (arrow). (**C**) Mild and diffuse infiltrate in the abdominal skin of a clinically affected dog (arrows). (**D**) Moderate and diffuse infiltrate in the ear skin of a clinically affected dog (arrows). (**E**) Severe and diffuse infiltrate in the ear skin of a clinically affected dog. HE staining. Images were adjusted only for brightness and contrast.

The animals were assessed based on clinical signs and histopathological features. The frequency of clinical signs observed in the 13 clinically affected dogs (group 2) were onychogryphosis (100%), dermatitis (92.3%), alopecia (76.9%), altered body condition (76.9%), lymphadenopathy (69.2%), and conjunctivitis (69.2%) (Supplementary Table S1). The inflammatory infiltrate showed two main patterns of distribution: localized primarily in the perivascular areas or diffuse throughout the dermis with different intensities (Figure 1A–E). We also observed periadnexal inflammatory infiltration. The ear skin showed more intense and more diffuse inflammatory infiltration than the abdominal skin (Table 1). Moreover, the ear skin of the clinically affected dogs showed a more diffuse inflammatory infiltrate than that of the subclinically affected dogs, whose inflammatory infiltrate was mainly located in the perivascular regions (Table 1).

Granulomatous reactions were also assessed in the ear and abdominal skin of both groups, and they occurred more frequently in the clinically affected dogs than in the subclinically affected dogs, were more apparent in ear skin fragments, and were associated with a higher intensity and distribution of inflammatory infiltrates (Table 2).

### 3.2. Ear Skin Showed a Higher Parasite Load than Abdominal Skin

**Figure 2:**
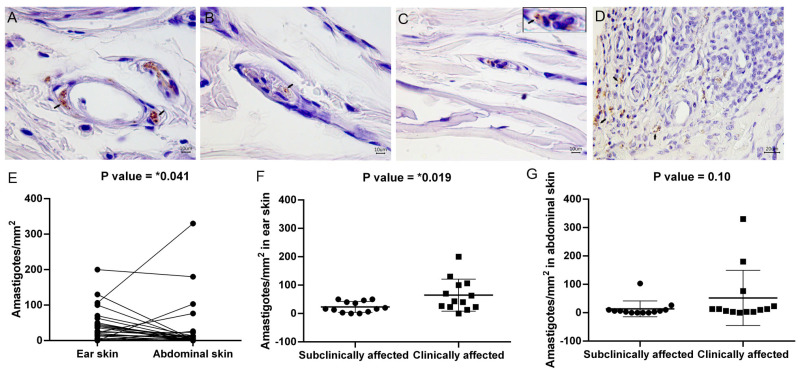
Qualitative and quantitative analyses of parasite load in the skin of dogs naturally infected with *L. infantum*. (**A**) *Leishmania* amastigotes parasitizing perivascular regions in the ear skin of a subclinically affected dog (arrows). (**B**,**C**) *Leishmania* amastigotes inside blood vessels in the ear skin of a subclinically affected dog. (**D**) *Leishmania* amastigotes diffusely distributed in the ear skin of a clinically affected dog (arrows). (**E**) Analysis of the number of amastigotes mm^2^ in paired samples of ear and abdominal skin (Wilcoxon test). (**F**) Amastigotes per mm^2^ in ear skin (unpaired *t* test). (**G**) Amastigotes per mm^2^ in abdominal skin (Mann-Whitney test). (**A**–**D**) Immunohistochemistry for *Leishmania* sp. Images were adjusted only for brightness and contrast. *p*-value summary: * significant.

To estimate whether there were differences in the number and distribution of amastigotes in different anatomical regions of the same dog, quantitative and qualitative analyses of the parasite load using an immunohistochemistry technique was conducted (Figure 2A–D). Amastigotes were observed parasitizing cells around blood vessels (Figure 2A) and inside blood vessels (Figure 2B,C) and were distributed in a diffuse manner in the skin (Figure 2D). The number of amastigotes/mm^2^ was higher in ear skin than in abdominal skin (Figure 2E). Moreover, the ear skin of the clinically affected dogs showed a higher parasite load than the ear skin of the subclinically affected dogs (Figure 2F). The abdominal skin showed no significant differences in the number of amastigotes/mm^2^ according to clinical status (Figure 2G) but showed an association between amastigote distribution and the intensity of inflammatory infiltrate (Table 3). No association was observed between the amastigote distribution and inflammatory infiltrate distribution in either region (Table 3).

### 3.3. Ear Skin Has a Greater Number of Blood Vessels and Larger Vessel Diameters than Abdominal Skin

**Figure 3:**
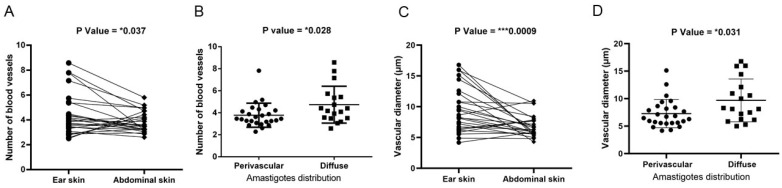
Association between *Leishmania* amastigote distribution and blood vessel amount and diameters in the skin of dogs naturally infected with *L. infantum*. (**A**) Comparative analysis of the number of blood vessels in paired samples of ear skin and abdominal skin of dogs naturally infected with *L. infantum* (Wilcoxon test, *p* = * 0.037). (**B**) Association between the number of blood vessels and the distribution of amastigotes (Mann-Whitney test). (**C**) Comparative analysis of vascular diameters in paired samples of ear skin and abdominal skin of dogs naturally infected with *L. infantum* (Wilcoxon test). (**D**) Association between vascular diameter and the distribution of amastigotes (Mann-Whitney test). *p*-value summary: * significant, *** extremely significant.

Due to the increase in the number and diameter of blood vessels seen during inflammatory processes, we examined whether there were differences in these parameters in different anatomical regions of the same dog infected with *L. infantum*. Blood vessel counts and vessel diameters were higher in ear skin than in abdominal skin (Figure 3A,C), mainly in clinically affected dogs (Supplementary Figure S1D,F). Furthermore, due to the observation of amastigotes within blood vessels, we examined whether the number and diameter of blood vessels were associated with the amastigote distribution. Skin samples with a greater number of blood vessels and larger vascular diameters also exhibited more diffuse amastigote distribution in the skin (Figure 3B,D). The association between amastigote distribution and a greater number of blood vessels was observed only in the ear skin (Supplementary Figure S1K).

### 3.4. Ear Skin Exhibited the Highest Number of Recently Infiltrating MAC387+ Cells

**Figure 4:**
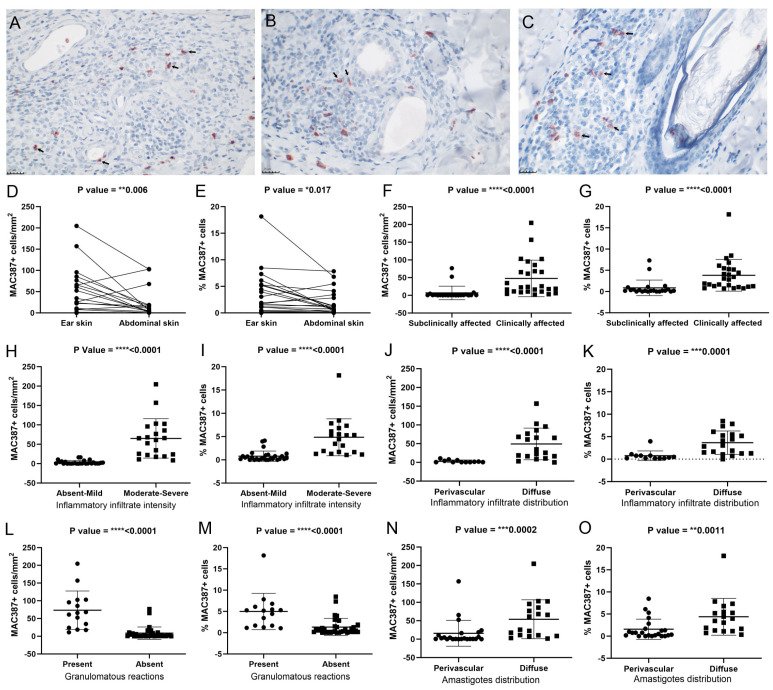
Analysis of MAC387+ cells (arrows) in the skin of dogs naturally infected with *L. infantum*. (**A**) Photomicrograph of cells labeled as MAC 387+ in the ear skin of a clinically affected dog. (**B**) Photomicrograph of cells labeled as MAC 387+ in the abdominal skin of a clinically affected dog. (**C**) Photomicrograph of cells labeled as MAC 387+ in the abdominal skin of a clinically affected dog. (**D**) Comparative analysis of MAC387+ cells/mm^2^ in paired samples of ear and abdominal skin (Wilcoxon test). (**E**) Comparative analysis of the percentage of MAC387+ cells in paired ear and abdominal skin samples (Wilcoxon test). (**F**) MAC387+ cells/mm^2^ according to clinical status (Mann-Whitney test). (**G**) Percentage of MAC387+ cells according to clinical status (Mann-Whitney test). (**H**) MAC387+ cells/mm^2^ according to the intensity of the inflammatory infiltrate (Mann-Whitney test). (**I**) Percentage of MAC387+ cells according to the intensity of the inflammatory infiltrate (Mann-Whitney test). (**J**) MAC387+ cells/mm^2^ according to the distribution of inflammatory infiltrates (Mann-Whitney test). (**K**) Percentage of MAC387+ cells according to the distribution of inflammatory infiltrates (Mann-Whitney test). (**L**) MAC 387+ cells/mm^2^ according to the presence of granulomatous reactions (Mann-Whitney test). (**M**) Percentage of MAC 387+ cells according to the presence of granulomatous reactions (Mann-Whitney test). (**N**) MAC 387+ cells/mm^2^ according to amastigote distribution (Mann-Whitney test). (**O**) Percentage of MAC 387+ cells according to amastigote distribution (Mann-Whitney test). (**A**–**C**) immunohistochemistry for MAC387. Images were adjusted only for brightness and contrast. *p*-value summary: * significant, ** very significant, *** or **** extremely significant.

Since we observed differences in the vasculature of the skin according to the anatomical region analyzed and the clinical status of the dog, we examined the cell influx based on the number of MAC387+ cells that had recently infiltrated the area. MAC387+ neutrophils and monocytes were found inside blood vessels, located between endothelial cells, and spread throughout the dermis (Figure 4A) adjacent to glands (Figure 4B) and hair follicles (Figure 4C). The ear skin exhibited a higher number of MAC387+ cells than the abdominal skin (Figure 4D,E). The clinically affected dogs showed a higher number of MAC 387+ cells than the subclinically affected dogs (Figure 4F,G). We assessed whether cell influx was associated with inflammatory infiltration and amastigote distribution. Skin samples with a higher number of MAC 387+ cells exhibited a more severe and diffuse inflammatory infiltrate (Figure 4H–K), a higher frequency of granulomatous reactions (Figure 4L–M), and a more diffuse distribution of amastigotes in the skin (Figure 4N–O). Only abdominal skin showed an association between the number of MAC387+ cells and amastigote distribution (Supplementary Figure S6C,D).

### 3.5. Ear Skin Exhibited an Increased Number of Vascular Endothelial Growth Factor (VEGF)+ Cells Compared to Abdominal Skin

**Figure 5:**
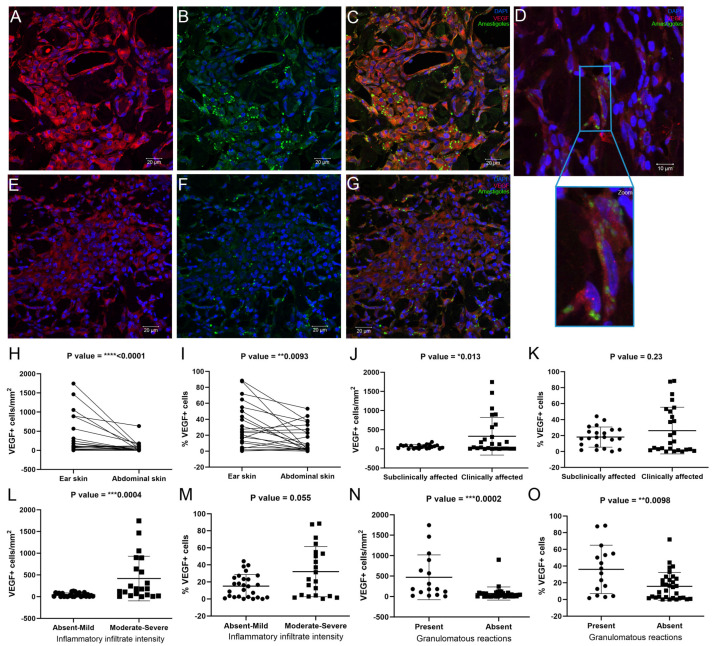
Analysis of VEGF+ cells in the ear and abdominal skin of dogs naturally infected with *L. infantum*. (**A**) Photomicrograph of ear skin showing VEGF+ cells (red). (**B**) Photomicrograph of ear skin showing amastigotes (green). (**C**) Merged image of (**A**,**B**). (**D**) Photomicrograph of ear skin showing amastigotes (green) parasitizing endothelial cells immunolabeled for VEGF (red). (**E**) Photomicrograph of abdominal skin showing VEGF+ cells (red). (**F**) Photomicrograph of abdominal skin showing amastigotes (green). (**G**) Merged image of (**E**,**F**). (**H**) VEGF+ cells/mm^2^ in paired samples of ear skin and abdominal skin (Wilcoxon test). (**I**) Percentage of VEGF+ cells in paired samples of ear and abdominal skin (Wilcoxon test). (**J**) VEGF+ cells/mm^2^ according to clinical status (unpaired *t* test). (**K**) Percentage of VEGF+ cells according to clinical status (unpaired *t* test). (**L**) VEGF+ cells/mm^2^ according to the intensity of the inflammatory infiltrate (Mann-Whitney test). (**M**) Percentage of VEGF+ cells according to the intensity of the inflammatory infiltrate (Mann-Whitney test). (**N**) VEGF+ cells/mm^2^ according to the presence of granulomatous reactions (Mann-Whitney test). (**O**) Percentage of VEGF+ cells according to the presence of granulomatous reactions (Mann-Whitney test). (**A**–**G**) Immunofluorescence for VEGF with nuclei in blue. Images were adjusted only for brightness and contrast. *p*-value summary: * significant, ** very significant, *** or **** extremely significant.

Due to its significant role in vascular remodeling, the expression of VEGF was assessed. VEGF expression was found within different cells of the inflammatory infiltrate and endothelial cells (Figure 5). Additionally, amastigotes were observed inside VEGF+ endothelial cells (Figure 5D). The expression of VEGF was higher in the ear skin than in the abdominal skin (Figure 5H,I), and the clinically affected dogs exhibited more VEGF+ cells than the subclinically affected dogs (Figure 5J). Skin samples with higher expression of VEGF exhibited a more severe inflammatory infiltrate (Figure 5L) and a higher frequency of granulomatous reactions (Figure 5N,O), mainly in the ear skin (Supplementary Figure S4A,K,L). No association was noted between the expression of VEGF and the distribution of inflammatory infiltrates (Supplementary Figure S3) or amastigotes (Supplementary Figure S5).

### 3.6. NOS2 Expression Was Associated with the Intensity and Distribution of Inflammatory Infiltrates

**Figure 6:**
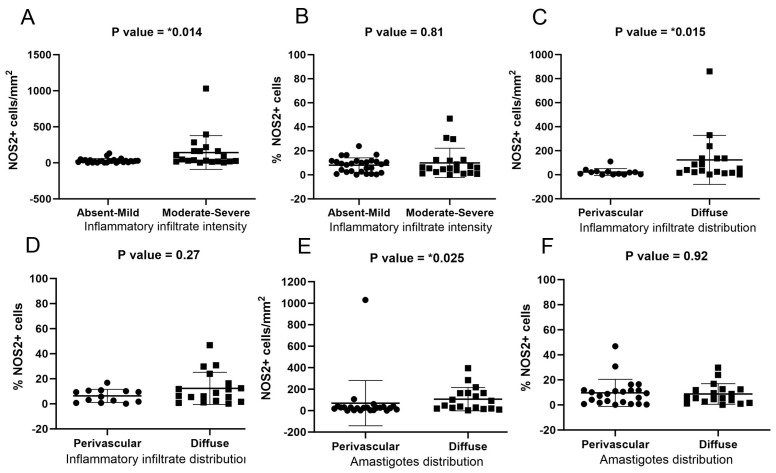
Analysis of NOS2+ cells in the ear and abdominal skin of dogs naturally infected with *L. infantum*. (**A**) NOS2+ cells/mm^2^ according to the intensity of the inflammatory infiltrate (Mann-Whitney test). (**B**) Percentage of NOS2+ cells according to the intensity of the inflammatory infiltrate (Mann-Whitney test). (**C**) NOS2+ cells/mm^2^ according to the distribution of inflammatory infiltrates (Mann-Whitney test). (**D**) Percentage of NOS2+ cells according to the distribution of inflammatory infiltrates (Mann-Whitney test). (**E**) NOS2+ cells/mm^2^ according to the distribution of amastigotes (Mann-Whitney test). (**F**) Percentage of NOS2+ cells according to the distribution of amastigotes (Mann-Whitney test). *p*-value summary: * significant.

NO molecules, a key component of the leishmanicidal response, is also implicated in angiogenesis and capillary dilation. Therefore, we evaluated whether there are differences in the expression of NOS2 in different regions of the dermis of the same dog and whether the expression of NOS2 is associated with the intensity and distribution of inflammatory infiltrates. No difference in NOS2 expression was observed during the evaluation based on the location of the skin sample (Supplementary Figure S5A,B) or clinical status of the dog (Supplementary Figure S5C,D). However, higher NOS2 expression was found in areas where inflammatory infiltration was more severe (Figure 6A), mainly in the abdominal skin (Supplementary Figure S5G) and diffusely distributed (Figure 6C), mainly in the ear skin (Supplementary Figure S5I). Higher NOS2 expression was observed in the abdominal skin that exhibited granulomatous reactions (Supplementary Figure S4O). Moreover, higher NOS2 expression was found in areas where amastigotes were more diffusely distributed (Figure 6F), mainly in the abdominal skin (Supplementary Figure S6M).

### 3.7. Abdominal Skin Exhibited a Higher NOS2/MAC387 Ratio than Ear Skin

**Figure 7:**
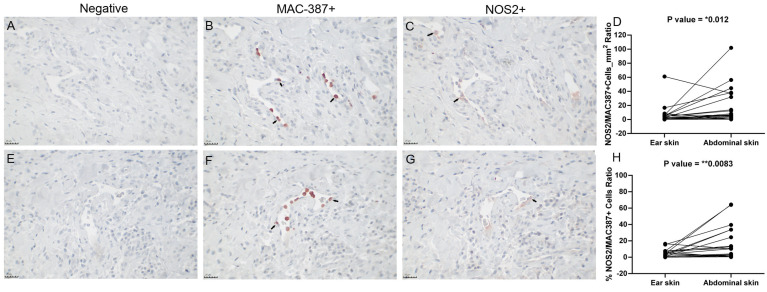
Comparative analysis of MAC387+ and NOS2+ cells in the skin of dogs naturally infected with *L. infantum*. (**A**) Negative control for images (**B**,**C**). (**B**) Photomicrograph of cells labeled for MAC 387 (arrows) in the ear skin of a subclinically affected dog. (**C**) Photomicrograph of cells labeled for NOS2 (arrows) in the same dog as image (**B**). (**D**) NOS2/MAC387/mm^2^ ratio in paired samples of ear and abdominal skin (Wilcoxon test). (**E**) Negative control for images (**F**,**G**). (**F**) Photomicrograph of cells labeled for MAC 387 in the ear skin of a subclinically affected dog. (**G**) Photomicrograph of cells labeled for NOS2 in the same dog as image (**F**). (**H**) NOS2/MAC387 ratio (%) of paired samples of ear and abdominal skin (Wilcoxon test). *p*-value summary: * significant, ** very significant.

The abdominal skin exhibited a higher expression of NOS2 when it exhibited a higher intensity of inflammatory infiltrate. Thus, we calculated the NOS2/MAC387+ cell ratio because the cell influx reflects the intensity of the inflammatory infiltrate and is responsible for the expression of this enzyme (Table 4). When the proportion of NOS2/MAC387+ cells was compared by skin region, the abdominal skin exhibited a higher relative NOS2 expression than the ear skin (Figure 7 and Table 4).

### 3.8. Ear Skin Exhibited Collagen Disruption Associated with Increased Intensity and Distribution of Inflammatory Infiltrate

**Figure 8:**
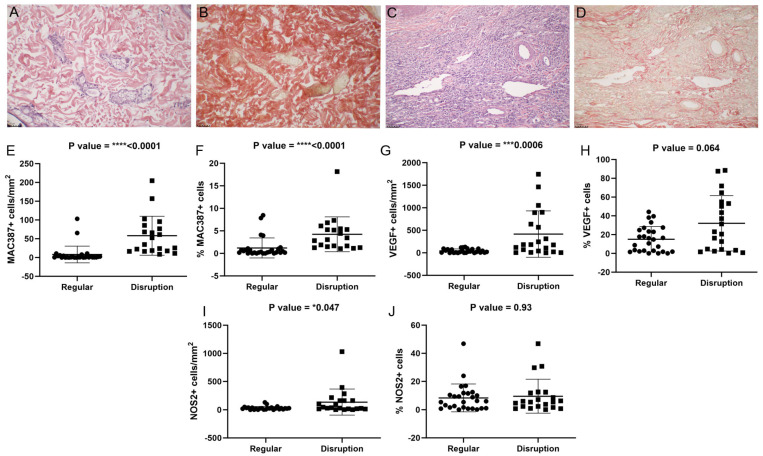
Association between collagen regularity and MAC387+, NOS2+, and VEGF+ cells in the ear and abdominal skin of dogs naturally infected with *L. infantum*. (**A**) Hematoxylin and eosin staining of the ear skin of a subclinically affected dog. (**B**) Picrosirius Red staining of the abdominal skin from the same dog as in image (**A**). (**C**) Hematoxylin and eosin staining of the ear skin of a clinically affected dog. (**D**) Picrosirius Red staining of the abdominal skin of the same dog as in image (**C**). (**E**) MAC387+ cells/mm^2^ (Mann-Whitney test). (**F**) Percentage of MAC387+ cells (Mann-Whitney test). (**G**) Expression of VEGF+ cells/mm^2^ (Mann-Whitney test). (**H**) Percentage of VEGF+ cells (Mann-Whitney test). (**I**) Expression of NOS2+ cells/mm^2^ (Mann-Whitney test). (**J**) Percentage of NOS2+ cells (Mann-Whitney test). Images were adjusted only for brightness and contrast. *p*-value summary: * significant, *** or **** extremely significant.

To assess the remodeling of collagen during the inflammatory process in the dermis of different regions, we labeled collagen in the ear and abdominal skin and verified its association with inflammatory infiltrate and parasite load. Collagen disruption was observed frequently in the ear skin of the clinically affected dogs. In the abdomen, all the subclinically affected dogs exhibited normal collagen deposition (Table 5). Most skin fragments with collagen disruption exhibited a higher intensity and distribution of inflammatory infiltrate (Table 5) (Figure 8A–D), and amastigotes were diffusely distributed in the skin (Table 6).

We investigated whether the cell influx and the expression of VEGF and NOS2 were associated with the disruption of collagen in the skin. Skin samples with collagen disruption exhibited a higher number of MAC387+, VEGF+, and NOS2+ cells (Figure 8E–J). A higher number of MAC387+ cells was associated with collagen disruption in both types of skin (Supplementary Figure S7A–D); when considering VEGF expression, only the ear skin showed an association with collagen disruption (Supplementary Figure S7I). For NOS2 expression, only the abdominal skin showed an association (Supplementary Figure S7G).

## 4. Discussion

Herein, we evaluated the inflammatory infiltrate in two distinct regions of the skin (ear and abdomen), the load and distribution of parasites in situ, and the organization of extracellular matrix collagen and studied the functionality of inflammatory cells through the expression of NOS2, MAC387, and VEGF. The choice of skin regions to be studied was based on anatomical differences in vascularization. In the present work, these anatomical vascular differences in the two regions of the skin were considered as parameters for the study of parasitic dissemination. We were able to observe amastigotes parasitizing endothelial cells and perivascular cells and showed that they were distributed throughout the tissue. The parasites were also observed inside blood vessels, indicating a possible route for dissemination of amastigotes through the skin via dermal blood vascularization. Using confocal microscopy, we demonstrated the colocalization of amastigotes and VEGF+ endothelial cells, which was demonstrated for the first time in dogs with visceral leishmaniasis. Studies of vascular changes have been carried out in other infection models, such as in the murine model of experimental infection in cutaneous leishmaniasis [14,22], but not for canine visceral leishmaniasis. Furthermore, the ear skin presented a more intense inflammatory process leading to an increase in the number and diameter of blood vessels, favoring the spread of the parasite from the blood to the dermis and vice versa. In this sense, the ear skin presented parasites in a greater quantity and a more dispersed pattern in the dermis, which was also previously demonstrated by other authors [7]. The differences observed between the ear and the abdomen constitute important findings, as they can help veterinarians during the diagnostic process, in which they can select a region of the skin that are more suitable for diagnostic methods, in addition to being less invasive. Thus, the expanded study of immunopathogenic mechanisms in the skin makes it possible to understand the dynamics of parasite distribution in a site accessible to the insect vector, contributing to the clinical management of infected dogs in endemic areas.

Dog skin is a strategic site for disease transmission since, regardless of the occurrence of clinical signs, it keeps viable parasites accessible to sandflies [23,24,25], with a parasitic load higher in those who are symptomatic [26,27]. *Leishmania* presents tropism for the skin, as demonstrated in dogs infected by vertical transmission and living in nonendemic areas [3]. Once in the skin, parasitic antigens stimulate the inflammatory process, leading to the recruitment of leukocytes to the site, with a relationship between the number of CD14+ monocytes in peripheral blood and the inflammatory manifestation in the skin [28]. In our study, we observed a relationship between local MAC387+ infiltrating leukocytes, the intensity of the inflammatory process, and the dispersion of parasites in the skin, mainly in the ear and in symptomatic animals. These data are similar to previous data showing that the skin of symptomatic dogs presents a higher parasite load associated with a more intense inflammatory infiltrate and a higher rate of apoptosis than asymptomatic animals [26,29]. This inflammatory infiltrate is composed of lymphocytes, plasma cells, mast cells, macrophages, and neutrophils [30,31,32]. Cells parasitized with amastigotes, such as neutrophils and macrophages, are frequently observed in inflammatory infiltration in the skin [29]. Therefore, we used a marker for MAC387+ infiltrating leukocytes (monocytes and neutrophils) associated with changes in the number and diameter of blood vessels as a way of studying the differences in cell influx into the skin of the ear and abdomen, which impact the spread of amastigotes through the dermis.

During inflammatory processes, blood vessels actively participate in the process of cell migration to injured tissues [33]. Increased vascular permeability, dilation of blood vessels, and recruitment of immune cells to the site of injury constitute important mechanisms of the inflammatory response [33]. Recently, infiltrated monocytes and macrophages can be identified using the marker MAC 387 [34]; however, in our study, in addition to monocytes and macrophages, recently infiltrated neutrophils were also found to be MAC 387+. In a murine model, early cell influx and persistence of neutrophils in ear skin after parasite transmission were important factors for the establishment and progression of the disease during infection by *L. major* [35]. In the present study, the ear skin showed a higher number of recently infiltrated cells than the abdominal skin. Altogether, the vascular and inflammatory changes observed in ear skin suggest a role for dermal vascularization in increasing the influx of inflammatory cells and possibly parasites into the tissue.

The increased influx of cells during inflammatory processes results in increased O2 demand and consequently results in low tissue oxygen levels [12]. To produce high levels of leishmanicidal NO, the induction of NOS2 is necessary, and low oxygen conditions reduce the production of NO by activated macrophages since these cells depend on the availability of O2 as a substrate to produce NO [12]. There was no difference in NOS2 expression when the ear and abdominal skin were compared; however, we noted that although the ear skin had a more intense level of inflammatory infiltrate than the abdominal skin, a relatively low level of NOS2 expression was observed. To analyze this finding, we calculated the NOS2/MAC387+ cell ratio since the influx of cells reflects the intensity of the inflammatory infiltrate and these cells are responsible for the expression of this enzyme. Notably, when the proportion of NOS2/MAC387+ cells was compared by skin region, the ear skin showed a lower expression of NOS2 than the abdominal skin despite showing a more intense level of inflammatory infiltrate. A possible explanation is that the inflammatory infiltrate in the ear skin had its function subverted by a high amount of parasites, becoming less capable of expressing NOS2 and, consequently, of controlling parasite growth. Other studies should be conducted to test this hypothesis. Conversely, the lower expression of NOS2 in the ear skin may be explained by the lack of available O2 resulting from the hypoxic environment generated by increased cell influx. Environments with low oxygenation may favor parasitic permanence in the tissue [12]. Because of local stimuli or low oxygenation, resident and/or infiltrating macrophages seemed to acquire an M2 profile (activated macrophages). Moreira et al. (2016) [36] showed that the number of M2 macrophages (CD163+ cells) in the ear skin of naturally infected dogs is associated with an increased parasite load [36]. An alternative activation of macrophages to an M2 profile also favors parasite replication [37]. Although there was no difference in NOS2 expression between ear and abdominal skin, associations between NOS2 expression and the intensity of the inflammatory infiltrate and the presence of granulomatous reactions were observed only in the abdominal skin, where the parasite load was generally lower. As NOS2 expression can be considered an M1 functional marker, this result suggests that in abdominal skin, in those samples with a high intensity of inflammatory cells, macrophages may be directed more toward an M1 profile compared to those in ear skin. Further experiments must be performed to understand the mechanism involved in this phenomenon. The association between parasite distribution and abundance of MAC387+ cells only in abdominal skin would suggest that the inflammatory infiltrate responds accordingly to the presence of amastigotes, both of which are more restricted in perivascular regions when the parasite load is low. 

Low tissue oxygen conditions are related to VEGF expression [12]. In our study, the ear skin showed higher levels of VEGF than the abdominal skin. VEGF can promote vascular dilation in vitro in a dose-dependent manner [13]. Our results indicate a role for VEGF in the vascular proliferation and dilation of blood vessels in the skin types evaluated, especially in the ear skin. Another role of VEGF is the induction of fenestrations between endothelial cells, increasing vascular permeability and facilitating the migration of cells from blood vessels to tissues [38]. In ear skin, VEGF may play a role in vascular remodeling, which would provide greater cell influx in this region, as observed in this study. The same effect was not observed in the abdominal skin, which had lower levels of VEGF and lower numbers of MAC387+ cells. In vitro studies by Lai et al. (2019) [39] described a three-way relationship between M2 macrophages, VEGF levels, and PDL-1 levels. Thus, VEGF is able to stimulate the polarization of macrophages to an M2 profile [39]. Furthermore, M2 macrophages are able to express PDL-1 under autocrine VEGF regulation, thereby participating in T-cell immunomodulation [39]. Due to the role of VEGF in macrophage polarization, the higher expression of VEGF in ear skin may influence the regulation of macrophages to an M2 profile in this region, resulting in increased parasite persistence. The observation of a parasite burden in cells expressing VEGF could indicate the favoring of parasite survival in these cells.

Another important factor associated with parasite dissemination and persistence in the skin is changes in dermal collagen fibers, which may favor the migration of parasitized macrophages into the blood vessels [7]. Thus, we performed an analysis of dermal collagen disruption. We observed that the abdominal skin showed lower collagen disruption than the ear skin. We observed an association between collagen disruption and amastigote distribution. This association indicates that in skin with higher collagen disruption, the distribution of the parasites in the tissue is facilitated. We assessed whether there was an association between collagen disruption and recently infiltrated MAC387+ cells. We were able to observe an association between the two parameters together and the location of the skin. The secretion of matrix metalloproteinases (MMP-9, MMP-3, MMP-2) by local and infiltrating cells and parasites plays an important role in the degradation of the extracellular matrix [7,40]] and in increasing cell migration into the tissue [38]. In the skin of dogs with visceral leishmaniasis, increased levels of matrix metalloproteinases, especially MMP-9, have been observed [7]. This justifies the increase in migrating cells in regions with disrupted dermal collagen. Taken together, these results suggest an important participation of dermal vascularization in parasite dissemination, mainly in ear skin. In addition to the hematogenous spread observed, the increased cell influx in ear skin in association with high levels of VEGF may result in a macrophage-rich environment polarized to an M2 profile, resulting in increased parasite dissemination and persistence in this region. The ear skin is an area that is more exposed to phlebotomine sandfly bites and other ectoparasites than abdominal skin. This constant stimulation may promote a basal microenvironment predisposed to polarization to a Th2 immune response. As reviewed by Arumugam et al. (2022) [41], parasites show high persistence at the primary bite site. Additionally, the secretions of sandfly salivary glands may modulate the immune response, favoring parasite persistence [42]. Thus, exposure to multiple bites could favor an increase and persistence of the parasite load. The abdominal skin is a region less exposed to sandfly bites, and the ratio of NOS2 to MAC387+ cells in the abdominal skin indicates a more effective response against the parasite, in which recently infiltrated immune cells are directed toward an M1 profile. Although the ear skin seems to be a potential site for vector infection, other studies should be conducted to better test the hypothesis described above.

## 5. Conclusions

The differences between the number of vessels and vascular diameters and the expression of VEGF in different regions of the skin of the same dog indicate that vascular alterations occur in different ways depending on the region being analyzed. A higher parasite load in the ear skin was associated with greater vascular changes in this region. Thus, vascular changes in the skin of dogs with CVL may play a role in the influx of cells from the vessels into the tissue and in parasite dissemination. The ear skin showed greater immunopathological changes associated with a high load and wide distribution of amastigotes, which makes it a choice site for diagnostic sampling, as well as the region most likely to serve as a source of parasites for the insect vector compared to the abdominal skin.

## Figures and Tables

**Table 1:** Frequency of the intensity and distribution of inflammatory infiltrates in the ear and abdominal skin in subclinically affected and clinically affected dogs naturally infected with *L. infantum*.

Skin Sample	Clinical Signs	Intensity of Inflammatory Infiltrate		*p* Value	Distribution of Inflammatory Infiltrate ^#^		*p* Value
		Absent—Mild	Moderate—Severe		Perivascular	Diffuse	
Ear skin	Subclinical	10	3	*** 0.001	7	3	** 0.007
	Clinically affected	1	12		1	10	
**Total**		11	15		8	13	
Abdominal skin	Subclinical	13	0	* 0.014	3	0	0.069
	Clinically affected	7	6		3	7	
**Total**		20	6		6	7	

^#^ Only animals showing inflammatory infiltration are represented. Fisher’s exact test. *p*-value summary: * significant, ** very significant, *** extremely significant.

**Table 2:** Frequency of the intensity and distribution of inflammatory infiltrates according to the presence or absence of granulomatous reaction in subclinically and clinically affected dogs naturally infected with *L. infantum*.

Skin Sample	Presence of Granulomatous Reaction	Intensity of Inflammatory Infiltrate ^#^		*p* Value	Distribution of Inflammatory Infiltrate		*p* Value
Ear skin		Absent—Mild	Moderate—Severe		Perivascular	Diffuse	
	Present	0	10	*** 0.0007	0	6	* 0.045
	Absent	11	5		8	7	
**Total**		11	15		8	13	
Abdominal skin		Absent—Mild	Moderate—Severe		Perivascular	Diffuse	
	Present	0	6	**** <0.0001	0	3	0.192
	Absent	20	0		6	4	
**Total**		20	6		6	7	

Fisher’s exact test. ^#^ Only animals showing granulomatous reactions are represented. *p*-value summary: * significant, *** or **** extremely significant.

**Table 3:** Frequency of the intensity of the inflammatory infiltrate and distribution of inflammatory infiltrate according to the amastigote distribution in subclinically and clinically affected dogs naturally infected with *L. infantum*.

Skin Sample	Amastigote Distribution ^#^	Intensity of Inflammatory Infiltrate		*p* Value	Distribution of Inflammatory Infiltrate		*p* Value
Ear skin		Absent—Mild	Moderate—Severe		Perivascular	Diffuse	
	Perivascular	6	5	0.21	3	4	0.62
	Diffuse	3	9		3	8	
**Total**		9	14		6	12	
Abdominal skin		Absent—Mild	Moderate—Severe		Perivascular	Diffuse	
	Perivascular	14	1	** 0.017	5	3	0.18
	Diffuse	1	5		0	3	
**Total**		15	6		5	6	

Fisher’s exact test. ^#^ Only animals showing amastigotes are represented. *p*-value summary: ** very significant.

**Table 4:** Values of %NOS2, %MAC387, and NOS2/MAC387 ratio based on skin region. Representation of mean, minimum, and maximum values according to skin sample.

	%NOS2	%MAC 387	NOS2/MAC387 Ratio **
**Ear skin**	5.450 (0.1000–46.84)	1.785 (0.0–18.15)	4.155 (0.0190–16.40)
**Abdominal skin**	10.31 (0.3600–30.75)	0.7800 (0.0–7.840)	10.483 (0.2600–64.44)
	NOS2/mm^2^	MAC 387/mm^2^	NOS2/MAC387 Ratio *
**Ear skin**	25.50 (0.5000–1030)	20.67 (0.0–135.1)	3.425 (0.0200–61.10)
**Abdominal skin**	31.35 (0.5000–217.5)	3.775 (0.0–86.22)	6.300 (0.1800–102.0)

Wilcoxon test. ** *p* value 0.0083; * *p* value 0.012.

**Table 5:** Frequency of collagen distribution patterns based on skin sample, clinical signs, and intensity and distribution of inflammatory infiltrates in subclinically and clinically affected dogs naturally infected with *L. infantum*.

Skin Sample	Variable	Classification	Distribution of Collagen		*p* Value
			Regular	Disruption	
**Ear**	Clinical signs	Subclinical	10	3	** 0.0048
		Clinically affected	2	11	
	Inflammatory infiltrate intensity	Absent–Mild	11	0	**** <0.0001
		Moderate–Severe	1	14	
	Inflammatory infiltrate distribution	Perivascular	8	0	** 0.0010
		Diffuse	3	10	
**Abdomen**	Clinical signs	Subclinical	13	0	** 0.0052
		Clinically affected	6	7	
	Inflammatory infiltrate intensity	Absent–Mild	18	2	** 0.0018
		Moderate–Severe	1	5	
	Inflammatory infiltrate distribution	Perivascular	6	0	0.069
		Diffuse	3	4	

Fisher’s exact test. *p*-value summary: ** very significant, **** extremely significant.

**Table 6:** Association between amastigote distribution and collagen deposition in the dermis.

Distribution of Parasite Load in Dermis	Distribution of Collagen in the Dermis		*p* Value
	Regular	Disruption	
**Perivascular**	20	6	** 0.0019
**Diffuse**	5	13	

Fisher’s exact test. *p*-value summary: ** very significant.

## Data Availability

Raw data supporting the conclusions of this study are available from the authors upon request.

## Supplementary Materials

The following supporting information can be downloaded at: https://www.mdpi.com/article/10.3390/pathogens13050371/s1, Figure S1: Comparative analysis between amastigotes/mm^2^, amastigote distribution, vascular diameter, and number of blood vessels in the ear and abdominal skin of dogs naturally infected with *L. infantum*; Figure S2: Association between MAC 387+ cells and the intensity and distribution of inflammatory infiltrate and the presence of granulomatous regions in ear skin and abdominal skin of dogs naturally infected with *L. infantum*; Figure S3: Association between VEGF+ cells, intensity and distribution of inflammatory infiltrate, and the presence of granulomatous regions in ear and abdominal skin of dogs naturally infected with *L. infantum*; Figure S4: NOS2 expression and its association with inflammatory infiltrate in ear and abdominal skin of dogs naturally infected with *L. infantum*; Figure S5: Association between MAC 387+ cells, VEGF+ cells, and NOS2+ cells and the distribution of parasite load in ear and abdominal skin of dogs naturally infected with *L. infantum*; Figure S6: NOS2/MAC387 ratio in paired samples of ear and abdominal skin of dogs naturally infected with *L. infantum*; Figure S7: Association between collagen disruption and MAC387+, NOS2+, and VEGF+ cells in the ear and abdominal skin of dogs naturally infected with *L. infantum*; Table S1: Frequency of clinical signs evaluated in 13 clinically affected dogs and their respective clinical scores; Table S2: Representation of the mean and minimum and maximum values of the analyzed variables according to skin fragment location and clinical status; Table S3: Representation of the mean and minimum and maximum values of the analyzed variables according to skin fragment location.
