# Supplementary material for: Anatomical Vascular Differences and Leishmania-Induced Vascular Morphological Changes Are Associated with a High Parasite Load in the Skin of Dogs Infected with Leishmania infantum
Source: Pathogens. 2024 Apr 30;13(5):371. doi: 10.3390/pathogens13050371 (PMC11123845; doi:10.3390/pathogens13050371)
Supplement: Supplementary file 1 [file pathogens-13-00371-s001.zip › pathogens-2714016-supplementary.pdf]

pathogens MDPI

Supplementary Materials

Supplementary Table S1. Frequency of clinical signs evaluated in 13 clinically affected dogs and their respective clinical scores.

Number	General clinical signs	Dermatological Clinical Signs	Clinical score
105/09	Lymphadenomegaly (1) loss of body condition (3)	Alopecia (2) Onychogryphosis (3)	9
113/09	loss of body condition (2) Lymphadenomegaly (2)	Alopecia (2) dermatitis (1) Onychogryphosis (1)	8
124/09	Ophthalmic alterations (3)	Alopecia (3) dermatitis (3) Onychogryphosis (3)	12
127/09	loss of body condition (2)	Alopecia (2) Onychogryphosis (3) dermatitis (3)	10
241/10	Lymphadenomegaly (1) loss of body condition (2) Ophthalmic alterations (3)	Dermatitis (1) Onychogryphosis (3)	10
243/10	loss of body condition (3)	Alopecia (1) dermatitis (1) Onychogryphosis (3)	8
253/10	loss of body condition (1) Lymphadenomegaly (1) Ophthalmic alterations (3)	Alopecia (1) dermatitis (3) Onychogryphosis (2)	11
254/10	Ophthalmic alterations (1) loss of body condition (2)	Dermatitis (2) Onychogryphosis (3) and ulcers	8
255/10	Ophthalmic alterations (1) Lymphadenomegaly (1) loss of body condition (2)	Alopecia (2) dermatitis (3) Onychogryphosis (1) and ulcers	10
259/10	loss of body condition (2) Lymphadenomegaly (2) Ophthalmic alterations (3)	Alopecia (2) dermatitis (2) Onychogryphosis (3)	14
261/10	Ophthalmic alterations (1) Lymphadenomegaly (3)	Alopecia (2) dermatitis (3) Onychogryphosis (2)	11
262/10	Ophthalmic alterations (1) Lymphadenomegaly (2) loss of body condition (3)	Alopecia (1) dermatitis (2) Onychogryphosis (3)	12
265/10	Lymphadenomegaly (2) Ophthalmic alterations (3)	Dermatitis (1) Onychogryphosis (3)	9

* Subclinical animals were not included in this table since they had no visible clinical signs (clinical score = 0).

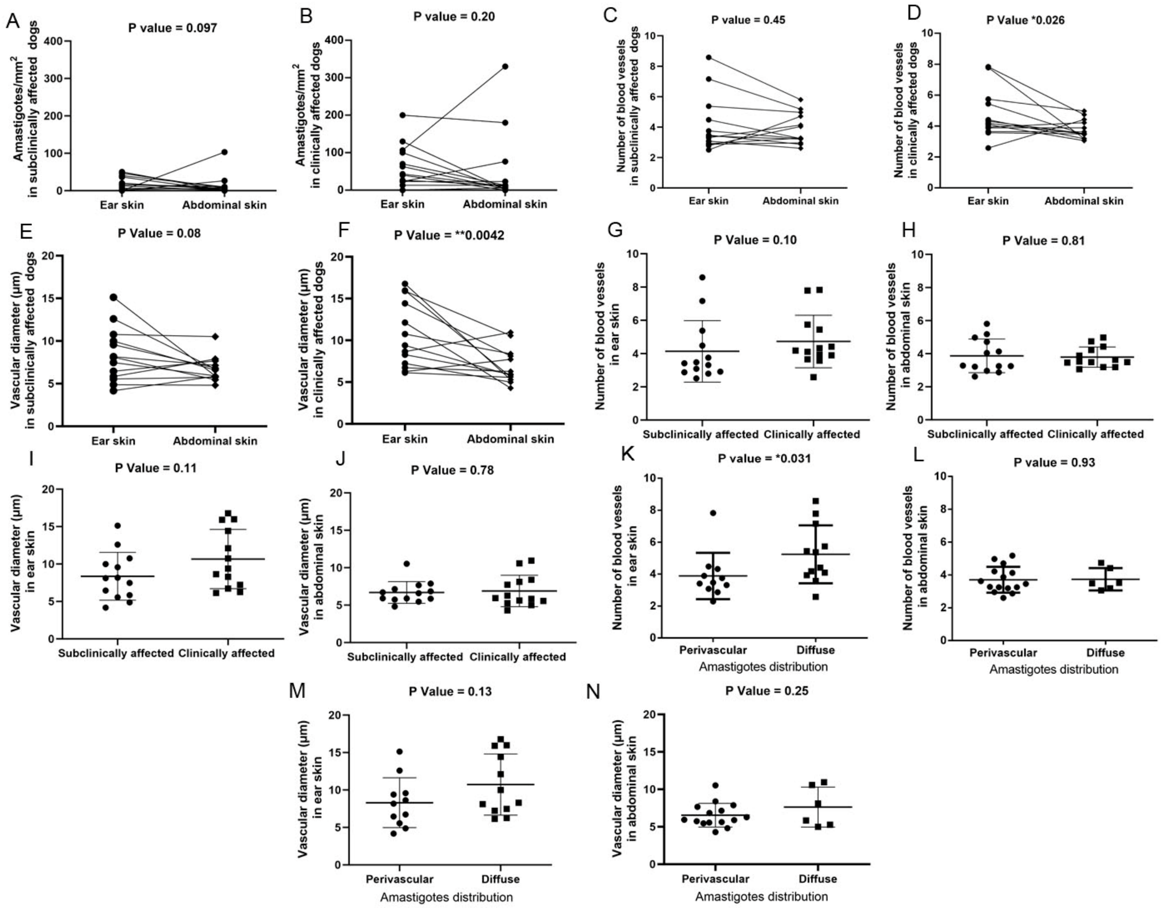

Supplementary Figure S1. Comparative analysis between amastigotes/mm^2, amastigote distribution, vascular diameter and number of blood vessels in the ear and abdominal skin of dogs naturally infected with *L. infantum*. A: Amastigotes/mm^2 in paired samples of ear and abdominal skin of subclinically affected dogs (Wilcoxon test. P = 0.097). B: Amastigotes/mm^2 in paired samples of ear skin and abdominal skin of clinically affected dogs (Wilcoxon test. P = 0.20). C: Number of blood vessels in paired samples of ear skin and abdominal skin of subclinically affected dogs (Wilcoxon test. P = 0.45). D: Number of blood vessels in paired samples of ear skin and abdominal skin of clinically affected dogs (Wilcoxon test. P = * 0.026). E: Vascular diameter in paired samples of ear skin and abdominal skin of subclinically affected dogs (paired t test. P = 0.08). F: Vascular diameter in paired samples of ear skin and abdominal skin of clinically affected dogs (paired t test. P = ** 0.008). G: Number of blood vessels in ear skin (Mann–Whitney test. P = 0.10). H: Number of blood vessels in the abdominal skin (unpaired t test. P = 0.81). I: Vascular diameter in ear skin (paired t test. P = 0.11). J: Vascular diameter in abdominal skin (unpaired t test. P = 0.78). K: Association between the number of blood vessels and amastigote distribution in ear skin (Mann–Whitney test. P = * 0.031). L: Association between the number of blood vessels and amastigote distribution in abdominal skin (unpaired t test. P = 0.93). M: Association between vascular diameter and amastigote distribution in ear skin (unpaired t test. P = 0.13). N: Association between vascular diameter and amastigote distribution in abdominal skin (unpaired t test. P = 0.25).

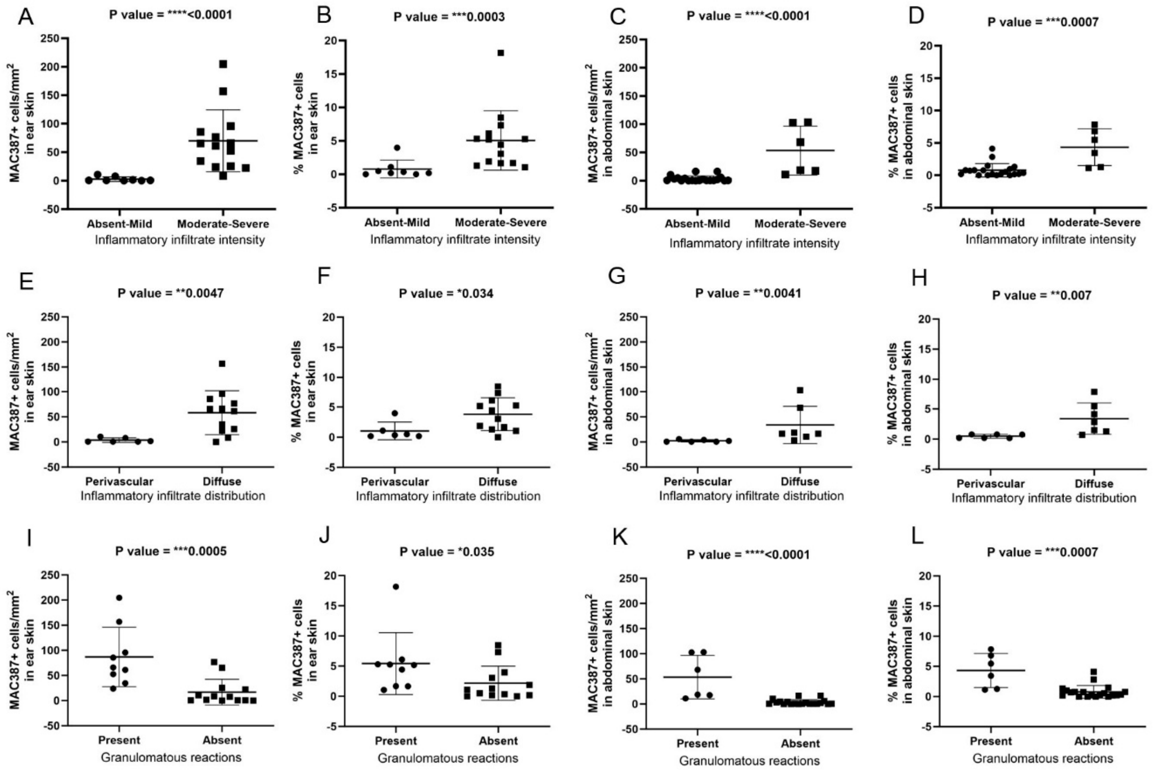

Supplementary Figure S2. Association between MAC 387 cells and the intensity and distribution of inflammatory infiltrate and the presence of granulomatous regions in ear skin and abdominal skin of dogs naturally infected with *L. infantum*. A: MAC387 cells/mm^2 according to the intensity of the inflammatory infiltrate in ear skin (Mann–Whitney test. P = **** < 0.0001). B: Percentage of MAC 387 cells according to the intensity of the inflammatory infiltrate in ear skin (Mann–Whitney test. P = *** 0.0003). C: MAC387 cells/mm^2 according to the intensity of the inflammatory infiltrate in abdominal skin (Mann–Whitney test. P = **** < 0.0001). D: Percentage of MAC 387 cells according to the intensity of the inflammatory infiltrate in abdominal skin (Mann–Whitney test. P = *** 0.0007). E: MAC387 cells/mm^2 according to the distribution of inflammatory infiltrate in ear skin (Mann–Whitney test. P = ** 0.0047). F: Percentage of MAC 387 cells according to the distribution of inflammatory infiltrate in ear skin (unpaired t test. P = * 0.034). G: MAC387 cells/mm^2 according to the distribution of inflammatory infiltrate in abdominal skin (Mann–Whitney test. P = ** 0.0041. H: Percentage of MAC 387 cells according to the distribution of inflammatory infiltrate in abdominal skin (Mann–Whitney test. P = ** 0.007). I: MAC 387 cells/mm^2 according to the presence of granulomatous regions in ear skin (Mann–Whitney test. P = *** 0.0005). J: Percentage of MAC 387 cells according to the presence of granulomatous regions in ear skin (Mann–Whitney test. P = * 0.035). K: MAC387 cells/mm^2 according to the presence of granulomatous regions in abdominal skin (Mann–Whitney test. P = **** < 0.0001). L: Percentage of MAC 387 cells according to the presence of granulomatous regions in abdominal skin (Mann–Whitney test. P = *** 0.0007).

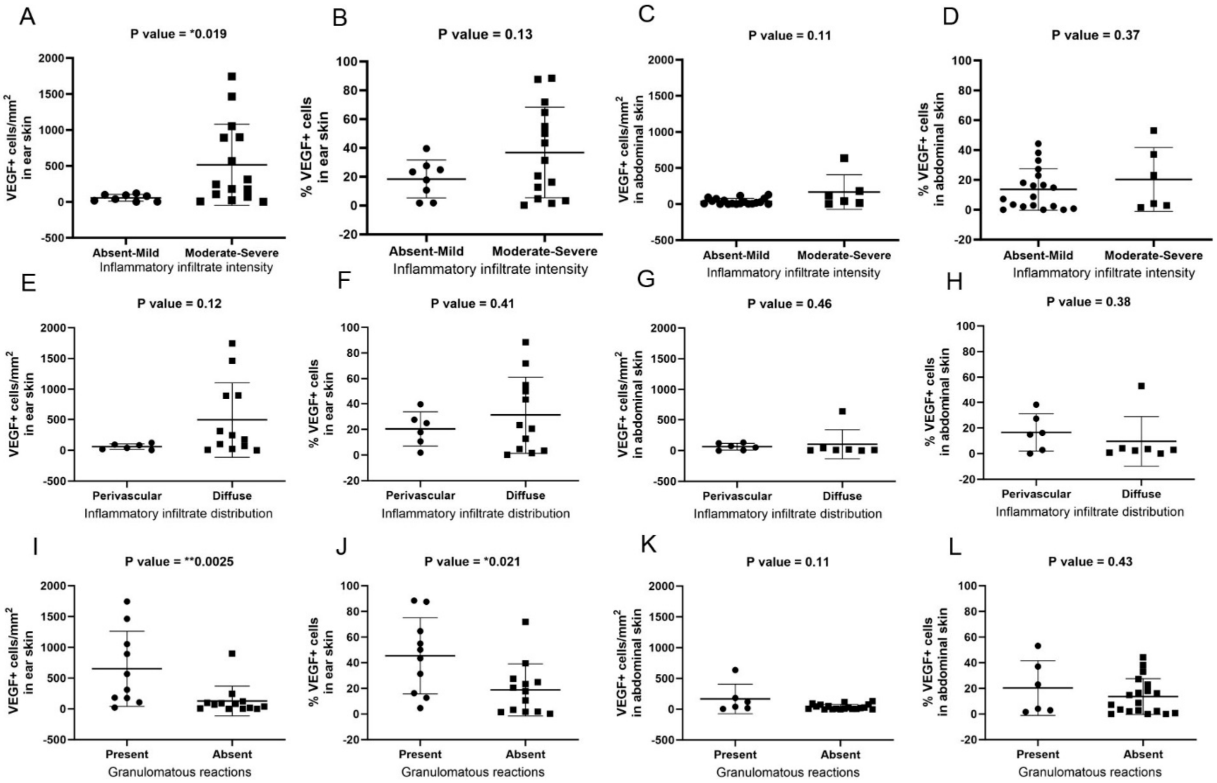

Supplementary Figure S3. Association between VEGF cells, intensity and distribution of inflammatory infiltrate, and the presence of granulomatous regions in ear and abdominal skin of dogs naturally infected with *L. infantum*. A: VEGF cells/mm^2 according to the intensity of the inflammatory infiltrate in ear skin (Mann–Whitney test. P = * 0.019). B: Percentage of VEGF cells according to the intensity of inflammatory infiltrate in ear skin (unpaired t test. P = 0.13). C: VEGF cells/mm^2 according to the intensity of the inflammatory infiltrate in abdominal skin (Mann–Whitney test. P = 0.11). D: Percentage of VEGF cells according to the intensity of the inflammatory infiltrate in abdominal skin (unpaired t test. P = 0.37). E: VEGF cells/mm^2 according to the distribution of inflammatory infiltrate in ear skin (Mann–Whitney test. P = 0.12). F: Percentage of VEGF cells according to the distribution of inflammatory infiltrate in ear skin (unpaired t test. P = 0.41). G: VEGF cells/mm^2 according to the distribution of inflammatory infiltrate in abdominal skin (Mann–Whitney test. P = 0.46). H: Percentage of VEGF cells according to the distribution of inflammatory infiltrate in abdominal skin (Mann–Whitney test. P = 0.38). I: VEGF cells/mm^2 according to the presence of granulomatous regions in ear skin (Mann–Whitney test. P = ** 0.0025). J: Percentage of VEGF cells according to the presence of granulomatous regions in ear skin (Mann–Whitney test. P = * 0.021). K: VEGF cells/mm^2 according to the presence of granulomatous regions in abdominal skin (Mann–Whitney test. P = 0.11). L: Percentage of VEGF cells according to the presence of granulomatous regions in abdominal skin (Mann–Whitney test. P = 0.43).

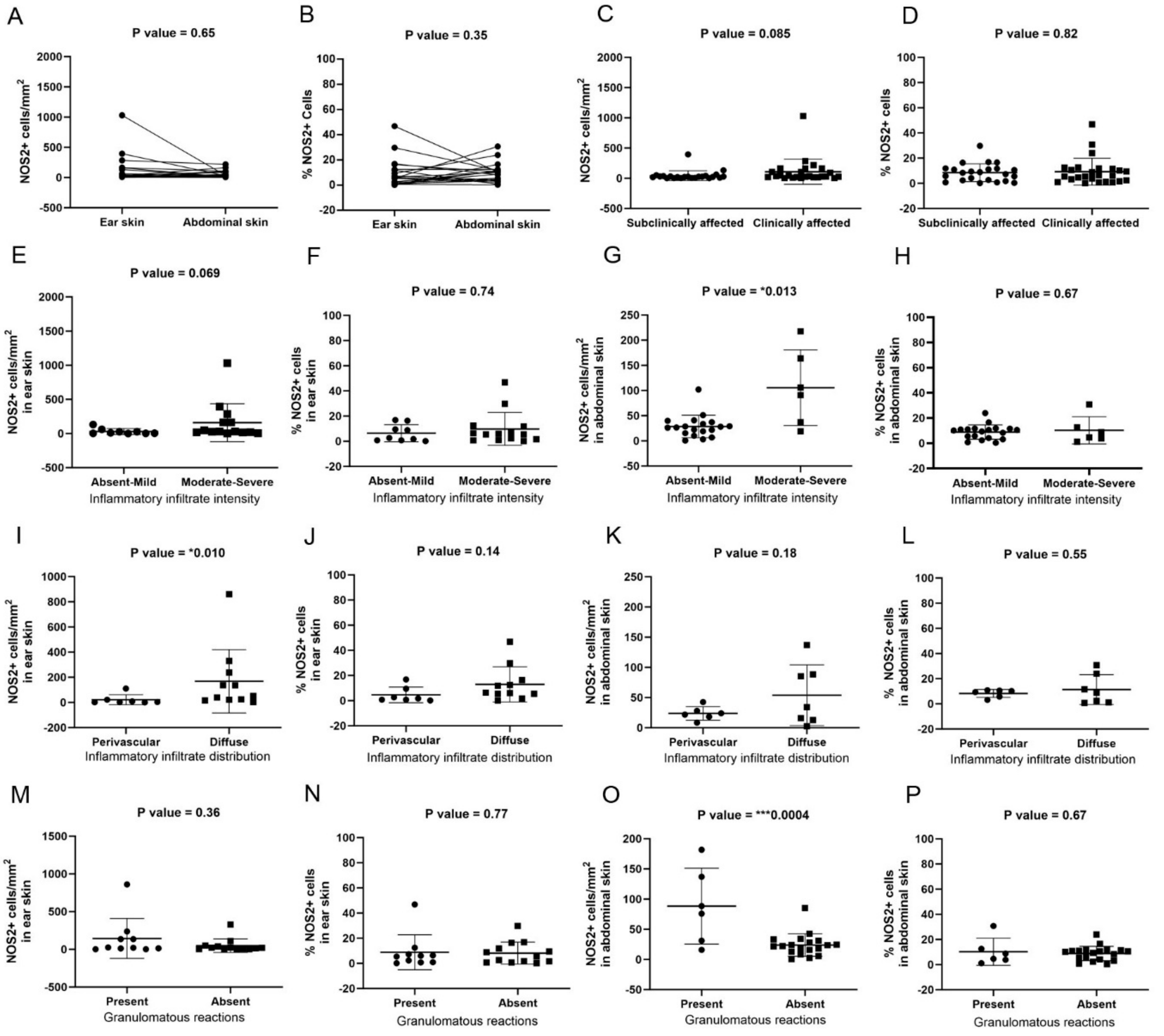

Supplementary Figure S4. NOS2 expression and its association with inflammatory infiltrate in ear and abdominal skin of dogs naturally infected with *L. infantum*. A: NOS2/mm^2 expression in paired samples of ear and abdominal skin (Wilcoxon test. P = 0.65). B: Percentage of NOS2 cells in paired samples of ear and abdominal skin (Wilcoxon test. P = 0.35). C: NOS2/mm^2 according to clinical status (Mann–Whitney test. P = 0.085). D: Percentage of NOS2 cells according to clinical status (Mann–Whitney test. P = 0.82). E: NOS2/mm^2 according to the intensity of inflammatory infiltrate in ear skin (Mann–Whitney test. P = 0.069). F: Percentage of NOS2 cells according to the intensity of inflammatory infiltrate in ear skin (Mann–Whitney test. P = 0.74). G: NOS2/mm^2 according to the intensity of the inflammatory infiltrate in abdominal skin (Mann–Whitney test. P = * 0.013). H: Percentage of NOS2 cells according to the intensity of inflammatory infiltrate in abdominal skin (Mann–Whitney test. P = 0.67). I: NOS2/mm^2 according to the distribution of inflammatory infiltrate in ear skin (Mann–Whitney test. P = * 0.010). J: Percentage of NOS2 cells according to the distribution of inflammatory infiltrate in ear skin (Mann–Whitney test. P = 0.14). K: NOS2/mm^2 according to the distribution of inflammatory infiltrate in abdominal skin (unpaired t test. P = 0.18). L: Percentage of NOS2 cells according to the distribution of inflammatory infiltrate in abdominal skin (unpaired t test. P = 0.55). M: NOS2 cells/mm^2 according to the presence of granulomatous regions in ear skin (Mann–Whitney test. P = 0.36). N: Percentage of NOS2 cells according to the presence of granulomatous regions in ear skin (Mann–Whitney test. P = 0.77). O: NOS2 cells/mm^2 according to the presence of granulomatous regions in abdominal skin (unpaired t test. P = *** 0.0004). P: Percentage of NOS2 cells according to the presence of granulomatous regions in abdominal skin (unpaired t test. P = 0.67).

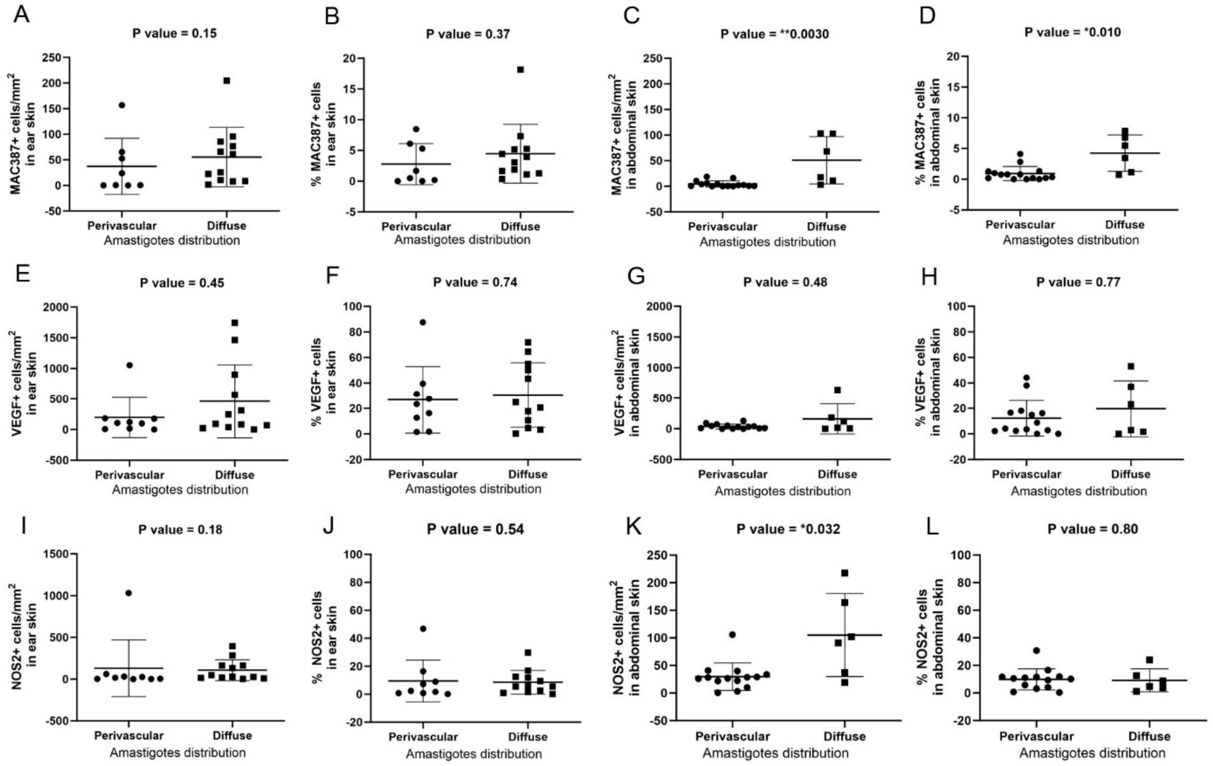

Supplementary Figure S5. Association between MAC 387 cells, VEGF cells and NOS2 cells and the distribution of parasite load in ear and abdominal skin of dogs naturally infected with *L. infantum*. A: MAC387 cells/mm^2 according to amastigote distribution in ear skin (Mann–Whitney test. P = 0.15). B: Percentage of MAC 387 cells according to amastigote distribution in ear skin (Mann–Whitney test. P = 0.37). C: MAC387 cells/mm^2 according to amastigote distribution in abdominal skin (Mann–Whitney test. P = ** 0.0030). D: Percentage of MAC 387 cells according to amastigote distribution in abdominal skin (Mann–Whitney test. P = * 0.010). E: VEGF cells/mm^2 according to amastigote distribution in ear skin (Mann–Whitney test. P = 0.45). F: Percentage of VEGF cells according to amastigote distribution in ear skin (unpaired t test. P = 0.74). G: VEGF cells/mm^2 according to amastigote distribution in abdominal skin (Mann–Whitney test. P = 0.48). H: Percentage of VEGF cells according to amastigote distribution in abdominal skin (Mann–Whitney test. P = 0.77). Figure I: NOS2 cells/mm^2 according to amastigote distribution in ear skin (Mann–Whitney test. P = 0.18). J: Percentage of NOS2 cells according to amastigote distribution in ear skin (Mann–Whitney test. P = 0.54). K: NOS2 cells/mm^2 according to amastigote distribution in abdominal skin (Mann–Whitney test. P = * 0.032). L: Percentage of NOS2 cells according to amastigote distribution in abdominal skin (Mann–Whitney test. P = 0.80).

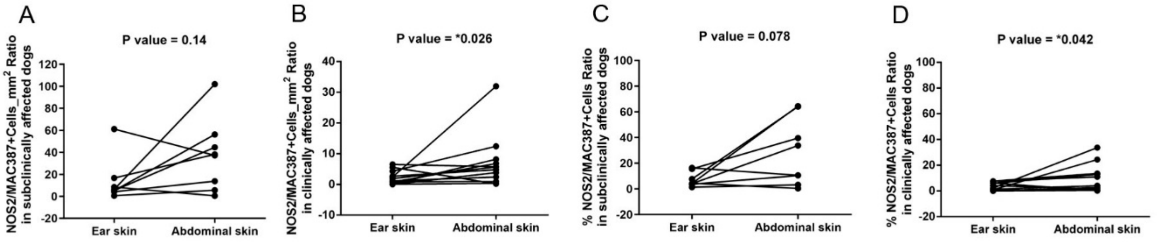

Supplementary Figure S6. **NOS2/MAC387 ratio in paired samples of ear and abdominal skin** of **dogs naturally infected with *L. infantum:*** A: NOS2/MAC387/mm^2 ratio in paired samples of ear and abdominal skin of subclinically affected dogs (Wilcoxon test. P = 0.14). B: NOS2/MAC387/mm^2 ratio in paired samples of ear and abdominal skin of clinically affected dogs (Wilcoxon test. P = * 0.026). C: % NOS2/MAC387 ratio in paired samples of ear and abdominal skin of subclinically affected dogs (Wilcoxon test. P = 0.078). D: % NOS2/MAC387 ratio in paired samples of ear and abdominal skin of clinically affected dogs (Wilcoxon test. P = * 0.042).

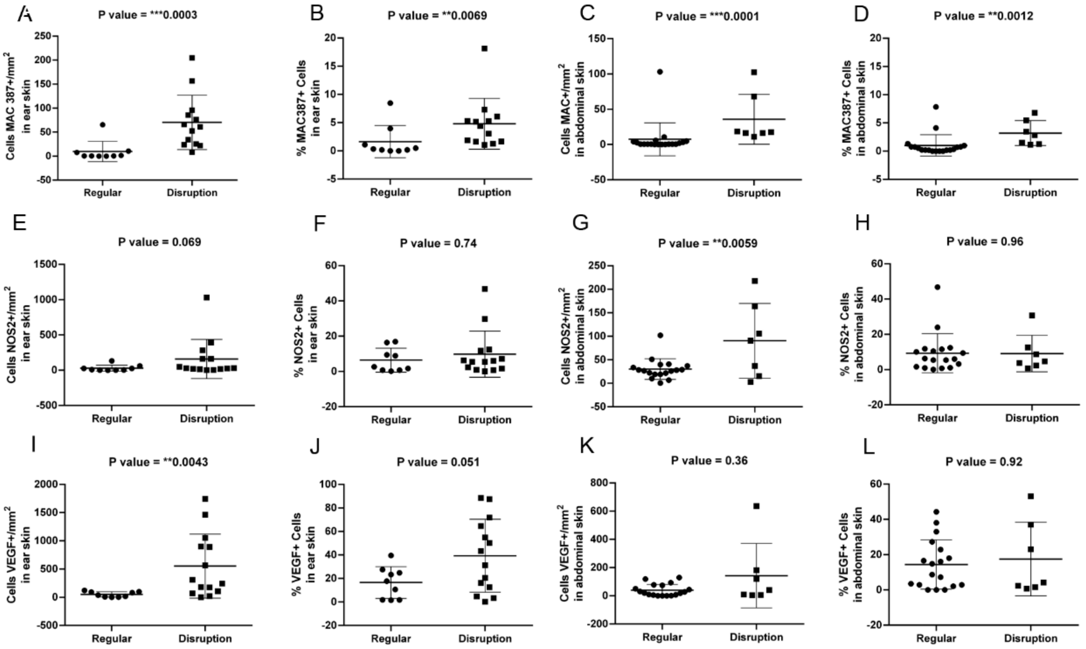

Supplementary Figure S7. Association between collagen disruption and MAC387, NOS2 and VEGF cells in the ear and abdominal skin of dogs naturally infected with *L. infantum*. A: MAC387 cells/mm^2 in ear skin (Mann–Whitney test. P = *** 0.0003) B: Percentage of MAC387 cells in ear skin (Mann–Whitney test. P = ** 0.0069). C: MAC387 cells/mm^2 in abdominal skin (Mann–Whitney test. P = *** 0.0001). D: Percentage of MAC387 cells in abdominal skin (Mann–Whitney test. P = ** 0.0012). E: NOS2 cells/mm^2 in ear skin (Mann–Whitney test. P = 0.069). F: Percentage of NOS2 cells in ear skin (Mann–Whitney test. P = 0.74). G: NOS2 cells/mm^2 in abdominal skin (unpaired t test. P =** 0.0059). H: Percentage of NOS2 cells in abdominal skin (Mann–Whitney test. P = 0.96). I: VEGF cells/mm^2 in ear skin (Mann–Whitney test. P = ** 0.0043). J: Percentage of VEGF cells in ear skin (unpaired t test. P = 0.051). K: VEGF cells/mm^2 in abdominal skin (Mann–Whitney test. P = 0.36). L: Percentage of VEGF cells in abdominal skin (Mann–Whitney test. P = 0.92).

Supplementary Table S2. Representation of the mean and minimum and maximum values of the analyzed variables according to skin fragment location and clinical status.

	**Subclinically affected**		**P Value**	**Clinically affected**		**P Value**	**P Value Subclinical and Clinically affected**
	**Ear skin**	**Abdominal skin**		**Ear skin**	**Abdominal skin**		
**Number of amastigotes**	23.07 (0-50.00)	13.59 (0-103.3)	0.097	64.61 (0-200.0)	52.05 (0-330.0)	0.20	* 0.041
**Number of blood vessels**	4.129 (2.500-8.580)	3.866 (2.610-5.800)	0.45	4.725 (2.580-7.830)	3.789 (3.060-4.970)	* 0.026	* 0.037

**Vascular diameter (µm)**	8.361 (4.176-15.13)	6.696 (4.814-10.53)	0.08	10.66 (6.141-16.77)	6.890 (4.289-10.95)	** 0.008	*** 0.0009

Supplementary Table S3. Representation of the mean and minimum and maximum values of the analyzed variables according to skin fragment location.

	Ear skin of Subclinically and Clinically affected dogs	Abdominal skin of Subclinically and Clinically affected dogs	P value
**Cells MAC 387/mm^2**	45.48 (0-204.7)	17.59 (0-103.2)	** 0.0066
**% MAC 387 Cells**	3.501 (0-18.15)	1.827 (0-7.840)	* 0.017
**Cells VEGF/mm^2**	372.5 (1.000-1745)	70.57 (0-636.1)	**** < 0.0001
**% VEGF cells**	31.68 (0.2500-88.46)	15.14 (0-53.11)	** 0.0093
**Cells NOS2/mm^2**	111.9 (0.5000-1030)	50.44 (0.5000-217.5)	0.65
**% NOS2 cells**	8.449 (0.1000-46.84)	9.715 (0.3600-30.75)	0.37
